# From sedentary volume to sedentary content in older inpatients with type 2 diabetes: a multicenter study

**DOI:** 10.3389/fpubh.2026.1907446

**Published:** 2026-08-20

**Authors:** Jing Cui, Na Zhang, Ju Ma, Yixiang Hu, Dongdong Wang, Xueyan Huang, Mengchi Li, Xinkun Wang, Wenhui Jiang

**Affiliations:** 1 School of Nursing, Health Science Center of Xi'an Jiaotong University, Xi'an, China; 2 School of Nursing, The Hong Kong Polytechnic University, Hong Kong, Hong Kong SAR, China; 3 Department of Operating Room, Xiangyang Central Hospital, Affiliated Hospital of Hubei University of Arts and Science, Xiangyang, China; 4 Department of Geriatrics, The First Affiliated Hospital of Guangxi Medical University, Nanning, China; 5 Department of Geriatrics, The Second People's Hospital of Gansu Province, Lanzhou, China

**Keywords:** older inpatients, physical function, screen-based sedentary content, sedentary behavior, type 2 diabetes

## Abstract

**Background:**

Sedentary behavior is common in older inpatients with type 2 diabetes mellitus (T2DM) and may coexist with poor metabolic control and functional decline.

**Objective:**

To identify factors associated with high sedentary time status and examine screen-based sedentary content in this population.

**Methods:**

This multicenter cross-sectional study recruited 419 older inpatients with T2DM from three tertiary hospitals in northwestern, central and southern China. Sedentary time and 10 types of sedentary content were assessed using a validated questionnaire. Personal, social, environmental and exercise-related psychological factors were obtained from medical records, field-based assessments and standardized instruments. A two-stage sequential modeling approach was used. Model I (*n* = 419) used hierarchical logistic regression to examine factors associated with high sedentary time status. Model II (*n* = 279) used binary logistic regression in the high-sedentary-time subgroup to examine factors associated with high screen tendency. The Mann–Whitney *U* test descriptively compared activity composition between participants in the high- and low-screen-tendency groups. Continuous-outcome sensitivity analyses assessed robustness to the operational classifications.

**Results:**

Of 419 participants, 279 (66.6%) were classified as having high sedentary time. Within this subgroup, 165 (59.1%) had a high proportion of screen-based sedentary time. High sedentary time status was associated with physical function, living context and exercise-related cognition. High screen tendency was characterized by age, postprandial glucose level, and living arrangement, gait speed showed only a category-specific contrast. Item-level analysis showed that participants with high screen tendency spent more time watching television, using computers and mobile phones, and less time chatting, eating, engaging in hobbies, napping and seated work. The Nagelkerke *R*^2^ values for Model I and Model II were 0.383 and 0.218, respectively. Continuous-outcome sensitivity analyses broadly supported the overall patterns.

**Conclusion:**

Sedentary behavior among older inpatients with T2DM was characterized not only by accumulated sedentary time but also by differences in sedentary content. These findings suggest that future sedentary behavior management could consider not only reducing total sedentary time but also identifying what patients do while sitting. Functional assessment, environmental support and strategies related to exercise outcome expectations and self-efficacy may represent potential intervention components that better reflect daily life, although their effects require confirmation in longitudinal and interventional studies.

## 1 Introduction

Sedentary behavior refers to any waking behavior performed in a sitting, reclining or lying posture with an energy expenditure of 1.5 METs or less (1, 2). Older adults show the highest levels of sedentary time (3) and have the highest diabetes prevalence across age groups, with type 2 diabetes mellitus (T2DM) representing 96% of all diabetes cases (4). China is experiencing rapid population aging and currently has the world’s largest number of diabetes cases, with a continuing upward trend (4). Older adults are projected to account for approximately 26% of the total population by 2050 (5). Given the combined background of population aging and diabetes burden, sedentary behavior is highly prevalent among older adults with T2DM in China. Previous studies reported an average sedentary time of approximately 7.01 ± 2.15 h per day, with 32.6% of patients spending at least 8 h per day sedentary (6). Another study reported that more than 80% of patients spent at least 6 sedentary hours each day (7). However, sedentary behavior has been linked to poor glycemic control (8), cognitive and physical functional decline, and an increased risk of hospitalization (9, 10), which may further increase the need for nursing and health care services (11). Therefore, reducing sedentary behavior is an important component of diabetes management (12), and identifying sedentary behavior profiles in older adults with T2DM has practical implications for healthcare.

Although reducing sedentary exposure has become an important goal in chronic disease management, several gaps remain in the current evidence. Previous studies on sedentary behavior have focused mainly on university students, office workers and older individuals in general (13–15), while evidence specific to older adults with T2DM remains limited. This population often faces poor metabolic control, reduced physical function and restrictions in daily activities, which may shape sedentary behavior differently from that in the general population (14). More importantly, most of the prior studies have used total sedentary time as the main indicator, with less attention given to the contexts and activity types in which sedentary behavior occurs (16). Because different sedentary activities may have different health implications, describing sedentary behavior only by total duration may limit the understanding of its internal structure and potential intervention targets (17).

In fact, sedentary activities differ in their content, including television viewing, mobile phone use, and reading or socializing (18). Although these activities are all low-energy behaviors, they differ in terms of social meaning, cognitive engagement and accompanying behaviors (19). For example, reading, cognitively engaging computer use or seated social interaction may be more mentally active, whereas prolonged television viewing may be more mentally passive (9). Evidence from China suggests that different sedentary contents may have different health implications for older adults with T2DM (20). Screen-related sedentary behavior may be more continuous, passive and individualized, whereas some nonscreen sedentary activities may occur in daily contexts such as meals, rest, communication or hobbies, with varying levels of cognitive engagement or interpersonal interaction (19, 21). Therefore, distinguishing sedentary behavior by both total volume and activity content may provide a more targeted basis for precise sedentary behavior interventions in older adults with diabetes (19).

Given that sedentary behavior involves not only time accumulation but also differences in activity content and context (22), research in this area should be grounded in a clear behavioral theory. Social Cognitive Theory (SCT) and the Theory of Planned Behavior (TPB) are commonly used to explain health behaviors in chronic disease management and have been increasingly applied to sedentary behavior and related cognitive processes (23, 24). SCT emphasizes the interaction between personal factors, the environment and behavior, and it can help explain how social support, the neighborhood environment and physical function are related to sedentary behavior (23, 25). TPB focuses on attitudes, outcome expectations and perceived behavioral control and helps explain how individuals form activity intentions and make daily behavioral choices (26). Therefore, this study integrated SCT and TPB to develop an analytical framework covering personal, environmental and behavioral cognitive factors to explain high sedentary time status and differences in sedentary content. On the basis of previous evidence and the theoretical constructs of SCT and TPB, the selected variables were grouped into three domains. Personal factors referred to demographic, clinical metabolic and physical function indicators. Environmental factors referred to social support and neighborhood environment. Behavioral cognitive factors referred to exercise outcome expectations and exercise self-efficacy.

However, existing studies on sedentary behavior among older adults with T2DM have focused primarily on total sedentary duration and have often been conducted in a single community or a single study setting, with relatively concentrated sample sources and life contexts (7). Therefore, this multicenter cross-sectional study analyzed high sedentary time status and sedentary content differentiation within an integrated SCT and TPB framework. The aim of the study was to identify factors associated with high sedentary time status among older inpatients with T2DM and to examine the proportion of sedentary time spent in screen-based activities among participants with high sedentary time. We further compared sedentary activity composition between participants with high and low screen tendency.

## 2 Methods

### 2.1 Design, setting and participants

This research followed a multicenter cross-sectional design and adopted nonprobability convenience sampling (27). It was carried out across three tertiary general hospitals in China. The multicenter design was intended to broaden the sample source, while convenience sampling was consistent with the recruitment conditions in real-world clinical settings. All the selected hospitals were high-level diabetes care centers with well-established specialist services and chronic disease management procedures.

Participants were eligible if they (1) were aged 60 years or older; (2) had a confirmed diagnosis of type 2 diabetes; (3) were conscious and able to communicate independently; and (4) had a basic ability to perform daily activities and voluntarily agreed to participate. The exclusion criteria were (1) cognitive impairment or communication difficulties and (2) mental disorders.

Based on the sample size estimation formula, $n=\frac{Z_{\alpha /2}^{2}\times \mathrm{pQ}}{d^{2}}$ , where *P* represents the estimated prevalence of sedentary behavior among older adults with T2DM in China. Based on previous literature, *p* = 80.37% (7), *Q* = 1-*P*, *α* was set at 0.05, $Z_{\alpha /2}\;$ = 1.96, and the allowable error was defined as *d* = 0.1*p*, namely, 8.04%. After a 20% sample loss was allowed, the minimum required sample size was estimated to be 113 participants. As this study used multicenter recruitment and planned to conduct two-stage logistic regression analyses, the sample size was expanded during recruitment, and 419 participants were ultimately included.

Eligible patients were screened by the nursing department at each center using electronic medical records and were invited to participate within 48 h of admission. Of the 419 participants, 151 were recruited from the central center, 143 from the western center and 125 from the southern center. The sedentary behavior questionnaire was used to ask participants to recall their usual sedentary activities in the home setting before admission. Written informed consent was obtained from all participants. Data collection occurred between March 2025 and September 2025 by a uniformly trained research team.

### 2.2 Measurement

Data were obtained from three sources: electronic medical records, field-based measurements and questionnaires. The measures included demographic characteristics, clinical and metabolic indicators, physical function, social and physical environments, exercise-related psychological factors and sedentary behavior. Authorization for scale use was granted by the original developers.

#### 2.2.1 Electronic health record information

Electronic health record information was extracted by research nurses at each center through the hospital information system (HIS). These data included demographic characteristics and clinical metabolic indicators. The demographic characteristics included age, gender, family history of diabetes, marital status, household income, living environment, living arrangement and education level. Clinical metabolic indicators included 2-h postprandial glucose (2hPG) and HbA1c, both of which were obtained from standardized laboratory tests at each center.

#### 2.2.2 Field-based measurement data

Field-based assessments were performed to evaluate anthropometric indicators and physical function. They were conducted by a uniformly trained research team in designated assessment areas at each center. Anthropometric indicators included height, weight and BMI. Physical function measures included handgrip strength, the five-time sit-to-stand test (5TCS), 6-m gait speed (6-m GS) and self-reported lower-limb strength.

1. Handgrip strength was used to evaluate upper-limb strength and assessed with a CAMRY EH101 digital dynamometer. The procedure followed Asian Working Group for Sarcopenia guidance (28) and referred to a standardized review on handgrip assessment in older populations (29). Participants were seated with both feet placed flat on the floor with the elbow bent to approximately 90°. Measurements were taken twice on the dominant side, and the highest value was retained as the final handgrip strength.
2. 5TCS was used to evaluate lower-limb strength and sit-to-stand ability. The test was timed using a YS 802C stopwatch, and the procedure followed the Short Physical Performance Battery (SPPB) protocol (30). Participants sat on a chair approximately 46 cm high, with their arms crossed over their chest, and were instructed to complete five sit-to-stand repetitions consecutively without using upper-limb assistance. The completion time was recorded. Participants who were unable to complete the test because of limited physical capacity were not treated as missing cases but were classified as “uncompleted” to retain information on functional limitations.
3. 6-m GS was used to assess mobility. The measurement procedure was consistent with that used for the 5TCS. Participants were instructed to walk 6 m at their habitual pace, and the completion time was recorded. The 5TCS and 6-m GS were categorized into relatively higher and lower performance groups according to median among participants who completed the corresponding assessment for exploratory analyses. Participants who were unable to complete either assessment were retained as a separate “uncompleted” category rather than treated as missing observations.
4. Subjective lower-limb weakness was recorded on the basis of participants’ self-reported lower-limb strength and was used to reflect their perceived lower-limb weakness.

#### 2.2.3 Questionnaire data

The questionnaire section included five validated standardized instruments to assess social support, the physical environment, exercise-related psychological factors and sedentary behavior.

1. The Social Support Rating Scale (SSRS) was used to assess social support. This instrument was developed by Xiao in 1986 for the Chinese (31). It contains 10 items across three dimensions: subjective support, objective support, and support utilization. Total scores range from 12 to 66, and higher scores indicate stronger social support. In the present study, Cronbach’s *α* reached 0.729.
2. The Chinese Physical Activity Neighborhood Environment Scale (PANES) was used to assess neighborhood environmental support for health behavior. The scale was refined by Sallis et al. (32) and adapted into Chinese by Xu et al. (33). It contains 17 items covering residential density, destination accessibility, neighborhood infrastructure, aesthetics, social environment, street connectivity, neighborhood safety and household motor vehicle ownership. In the present sample, Cronbach’s *α* reached 0.880.
3. Outcome Expectations for Exercise Scale (OEES) was used to assess exercise outcome expectations. The instrument was originally developed by Resnick et al. (34) and later translated into Chinese by Lee et al. (35). It contains 9 items, and the mean item score ranges from 1 to 5. Higher values reflect more positive exercise outcome expectations. In this study, Cronbach’s *α* was 0.955.
4. The Exercise Self-Efficacy Scale (ESE) was used to assess exercise self-efficacy. The instrument was developed by Benisovich et al. (36), and it contains 18 items across six dimensions: negative affect, making excuses, exercising alone, lack of equipment, resistance from others and bad weather. After permission was obtained, two bilingual nursing researchers translated and culturally adapted the scale. In the present sample, Cronbach’s *α* reached 0.965.
5. The Sedentary Behavior Questionnaire for the Older Adults (SBQ) was used to assess sedentary behavior. The SBQ was developed by Ku et al. (37) and contains 10 items that measure the duration of different sedentary activities during the previous 7 days. Total sedentary time was calculated by summing the average daily duration across the 10 SBQ items, consistent with the scoring framework of the original questionnaire. As the SBQ is a formative scale, its items represent different sedentary contexts rather than a single underlying construct. Cronbach’s *α* is therefore not appropriate for assessing its reliability (38, 39). Previous research has shown an acceptable intraclass correlation coefficient (ICC) for the SBQ (37). In this study, a 14-day test–retest assessment was conducted in a subsample (n = 40). The ICC for total sedentary time was 0.881 (*p* < 0.001), which indicated good temporal stability. Detailed item-level test–retest results are provided in Supplementary Appendix 1.

Participants were classified into the high sedentary time group (>6 h/day) and the low sedentary time group (≤6 h/day) using an operational threshold based on total sedentary time (7). Although sedentary behavior has been standardized conceptually, no universally accepted time-based threshold has been established for sedentary behavior classification (40). Previous epidemiological studies have applied a range of operational thresholds for prolonged sedentary exposure, with 6 h/day being one of the operational criteria used, particularly among older adults and populations with chronic conditions (41, 42). A similar criterion has also been used among older adults with T2DM in China, supporting its applicability to the population examined in the present study (7). The screen-based sedentary proportion was then computed as SBQ items 1 and 2 divided by total sedentary time. A value of 0.35, derived from the median screen-based sedentary proportion in the full sample, was used as a common operational reference among participants with high sedentary time. Participants with a proportion >0.35 were classified into the high screen tendency group, whereas those with a proportion ≤0.35 were classified into the low screen tendency group. High screen tendency indicates a study-specific operational category representing a relatively higher proportion of screen-based sedentary time among participants with high sedentary time, rather than a formally established behavioral phenotype, and does not mean that all sedentary activities were screen-based.

#### 2.2.4 Outcome definitions

Two outcome variables were defined in this study. The outcome for Model I was a binary indicator of total sedentary time, with participants classified according to whether their average daily sedentary time exceeded 6 h/day. The outcome for Model II was sedentary content differentiation, which was classified among participants with high sedentary time as high or low screen tendency groups according to the screen-based sedentary proportion. For the continuous-outcome sensitivity analyses, total sedentary time was retained in hours per day, and the screen-based sedentary proportion was retained as a continuous proportion ranging from 0 to 1. The independent variables were grouped into three categories on the basis of the theoretical framework. The personal factors included demographic, clinical metabolic and physical function indicators. The environmental factors included social support and physical environment indicators. Behavioral cognitive factors included outcome expectations for exercise and exercise self-efficacy.

#### 2.2.5 Data collection and quality control

All the questionnaires were distributed and completed on site under the guidance of research nurses. After completion, the data were entered independently by two researchers and checked for consistency.

### 2.3 Data analysis

All statistical analyses were performed using SPSS 26.0 and R 4.4.3. Before formal analysis, data cleaning was conducted, which included missing value analysis, normality testing and assessment of common method bias. Inability to complete the 5TCS or 6-m GS test was regarded as a meaningful functional status and retained as an “uncompleted” category. The missing rates for 2hPG and HbA1c were 0.2 and 3.8%, respectively, and the mean imputation was used. Given the low proportion of missing clinical data, a complete-case sensitivity analysis was additionally conducted for Model II to evaluate whether the observed associations were robust to alternative missing-data handling approaches. Harman’s single-factor test was applied to evaluate common method bias. Descriptive data were summarized using frequencies, percentages, means, standard deviations, medians and interquartile ranges. A sunburst chart was constructed to display the distributions of high sedentary time status and sedentary content differentiation.

The internal consistency of the SSRS, PANES, OEES and ESE was assessed using Cronbach’s *α*. As the SBQ is a formative scale, Cronbach’s α was not applicable. Therefore, the 14-day ICC was assessed in a subsample of 40 participants.

A two-stage sequential modeling approach was used. Model I examined high sedentary time status using theory-driven hierarchical logistic regression. Personal factors were entered as predefined covariates using the enter method. Environmental and behavioral cognitive factors subscale variables with *p* < 0.05 in the univariate analyses were included in the candidate set and subjected to a likelihood ratio-based stepwise procedure to obtain a parsimonious model while retaining theoretical interpretability. Model II examined factors associated with high screen tendency. Candidate variables were entered simultaneously using the Enter method to maintain model stability. The complete-case sensitivity analysis used the same candidate variables and modeling procedures as the primary Model II analysis.

To assess robustness to the operational cut-offs, three fixed-covariate continuous-outcome sensitivity analyses were conducted. Total sedentary time was analyzed in all 419 participants using multivariable linear regression, whereas the screen-based sedentary proportion was analyzed using fractional logistic regression among the 279 participants with high sedentary time and, additionally, in the full sample of 419 participants. All models used HC3 robust standard errors and simultaneous entry of covariates from the corresponding final categorical model, without additional variable screening; the categorical models remained the primary analyses.

Multicollinearity was checked using variance inflation factors, and VIF values less than 10 were considered acceptable. Influential observations were examined using Cook’s distance for the linear regression model and generalized Cook’s distance for the fractional logistic regression models. For multicategory variables, overall robust tests were also conducted. Differences in the time distribution of the 10 SBQ items were examined with the Mann–Whitney U test as a descriptive item-level analysis to compare the activity composition between participants with high and low screen tendencies.

### 2.4 Ethical considerations

The study was approved by the Ethics Committee of the lead institution, Xi’an Jiaotong University Health Science Center (No. LLSBPJ-2024-698), and by the ethics committees of the collaborating institutions, Xiangyang Central Hospital (No. 2025–113) and the First Affiliated Hospital of Guangxi Medical University (No. 2025-S0448). All methods were performed in accordance with the relevant guidelines and regulations, including the Declaration of Helsinki. Informed consent was obtained from all participants.

## 3 Results

### 3.1 Stratified distribution of high sedentary time status and screen-based content differentiation

As some variables relied on self-reports, common method bias was evaluated using Harman’s single-factor test. The largest factor accounted for 26.75% of the variance, which was below the 40% threshold (43), indicating a limited risk of common method bias.

**Figure 1:**
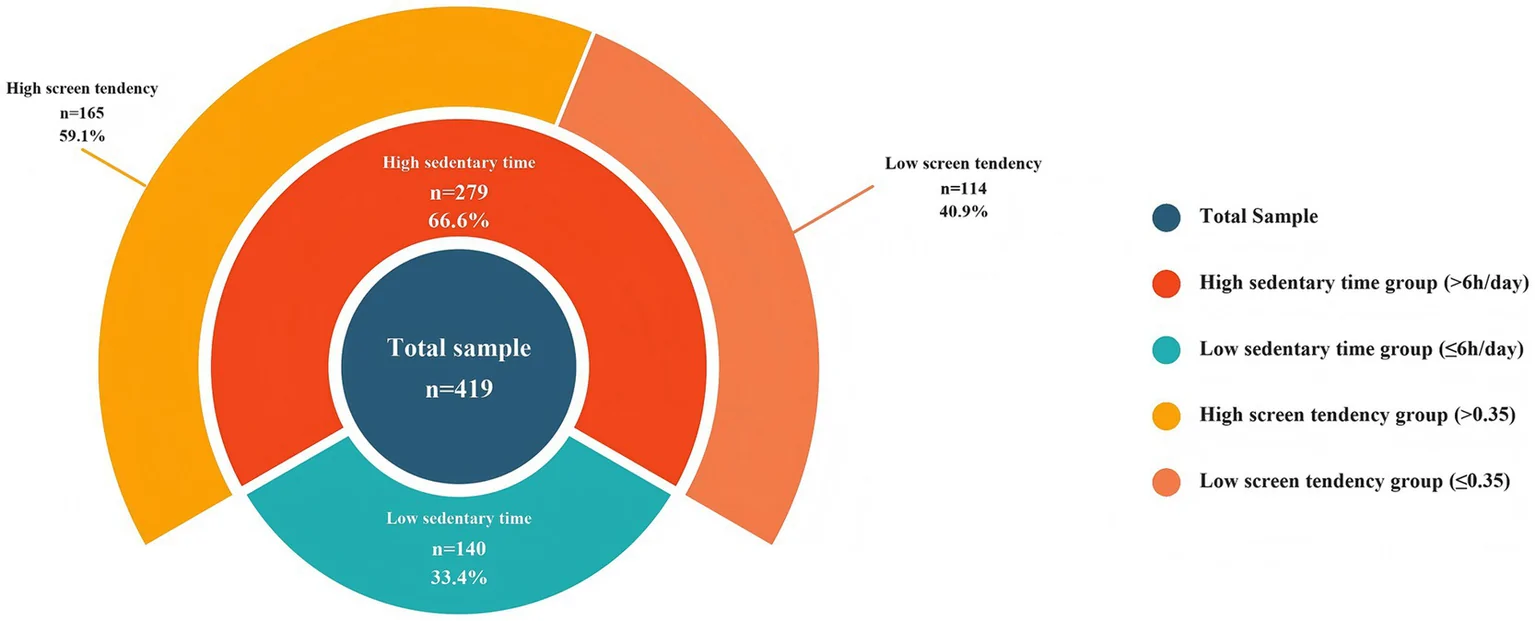
Sunburst chart of sample composition and stratified sedentary behavior among older inpatients with T2DM.

Overall, 419 older inpatients with T2DM were enrolled, and 279 (66.6%) were categorized as having high sedentary time. Among the participants with high sedentary time, 165 (59.1%) had a high screen tendency, and 114 (40.9%) had a low screen tendency. The stratified distributions of the total sample, high sedentary time status and screen tendency among participants with high sedentary time are shown in Figure 1. This structure corresponds to the subsequent high sedentary time status model (n = 419) and the sedentary content differentiation model among participants with high sedentary time (n = 279).

### 3.2 Descriptions of participants’ characteristics

The characteristics of the total sample are shown in Table 1. The mean age of the participants was 69.63 years (SD = 7.65). Most participants were married, lived with family members and resided in urban areas. Approximately 40% had a family history of diabetes. Overall, the sample was characterized by poor metabolic control and functional limitations. The levels of 2hPG and HbA1c were relatively high. Physical function also varied across participants. Approximately 40% of the participants were classified as having higher performance in the 5TCS and 6-m GS tests, whereas 15–17% were unable to complete the corresponding physical function tests.

**Table 1:** Participant characteristics (*n* = 419).

Variable	*n* (%)	(M ± SD)/M (IQR)	Missing *n* (%)
Age (years)		69.63 ± 7.65	0
BMI (kg/m^2^)		24.77 ± 3.92	0
2hPG		15.19 ± 5.56	1 (0.2)
HbA1c (%)		8.90 ± 2.25	16 (3.8)
Handgrip strength		22.88 ± 9.02	0
Five-times-sit-to-stand test (5TCS)		12.35 (9.50–16.00)	0
Higher performance	174 (41.5)		
Lower performance	173 (41.3)		
Uncompleted	72 (17.2)		
6-m gait speed (6-m GS)		0.88 (0.61–1.07)	0
Higher performance	176 (42.0)		
Lower performance	178 (42.5)		
Uncompleted	65 (15.5)		
Subjective lower-limb weakness			0
Yes	183 (43.7)		
No	236 (56.3)		
Gender			0
Male	214 (51.1)		
Female	205 (48.9)		
Family history of diabetes			0
Yes	180 (43.0)		
No	239 (57.0)		
Marital status			0
Unmarried	110 (26.3)		
Married	309 (73.7)		
Monthly household income (CNY)			0
<2000	107 (25.5)		
≥2000	312 (74.5)		
Education level			0
Primary school	100 (23.9)		
Junior high school	142 (33.9)		
Senior high school or above	177 (42.2)		
Living environment			0
Rural	85 (20.3)		
Urban	334 (79.7)		
Living arrangement			0
With spouse	242 (57.8)		
With children	130 (31.0)		
Alone or with others	47 (11.2)		

### 3.3 Univariate comparisons for high sedentary time status and screen-based content differentiation

To align with the modeling strategy, personal-level variables were entered as predefined covariates in the multivariable models. Therefore, Table 2 reports only the test statistics and *p* values for the behavioral and environmental factors. Full descriptive characteristics and univariate comparisons for high sedentary time status and screen-based content differentiation are provided in Supplementary Appendix 2. Group comparisons were conducted using independent-samples *t-*tests or χ^2^ tests.

**Table 2:** Univariate comparisons for high sedentary time status and screen-based content differentiation.

Item	High sedentary time comparison (*n* = 419)	Screen-based content differentiation comparison (*n* = 279)
SSRS		
Subjective support	−4.080***	1.062
Objective support	−1.653	0.600
Support utilization	−0.809	−0.300
PANES		
Destination accessibility	−0.795	0.775
Neighborhood infrastructure	−7.391***	1.605
Social environment	−4.329**	−0.411
Aesthetic value	−4.600***	−0.633
Street connectivity	−4.174***	0.165
Neighborhood safety	−3.892 ***	1.238
Residential density	3.499**	2.238*
Household motor vehicle ownership	−4.232***	−0.992
OEES	−7.072***	0.280
ESE		
Negative affect	−4.840***	0.726
Making excuses	−4.474***	1.602
Exercising alone	−3.919 ***	1.172
Lack of exercise equipment	−6.010***	2.160*
Resistance from others	−7.046 ***	0.678
Bad weather	−2.470*	0.265

*P < 0.05, **p < 0.01, ***p < 0.001.

The high sedentary time group generally had lower levels of exercise-related cognition and environmental support. Between-group differences were observed in the OEES mean score, several ESE dimensions, SSRS subjective support and several PANES dimensions. The differences were particularly evident for the OEES mean score, the ESE resistance from the others dimensions and the PANES neighborhood infrastructure dimension (all *p* < 0.001). Fewer differences were found between the high- and low-screen-tendency groups. Only the PANES residential density and the ESE lack of equipment dimension differed between groups (*p* < 0.05), suggesting that content differentiation was more related to specific environmental and barrier-related dimensions than to overall support levels.

### 3.4 Item-level comparison of sedentary activity composition between the high- and low-screen-tendency groups

To further describe the activity composition of the high- and low-screen tendency groups, the time distribution of the 10 SBQ items among participants with high sedentary time was examined with the Mann–Whitney U test. The findings are shown in Table 3. The high-screen-tendency group spent more sedentary time watching television or videos and using computers, the internet, mobile phones or games (both *p* < 0.001). Beyond these screen-based activities, this group spent less sedentary time chatting or talking on the phone, eating, engaging in hobbies, napping and seated work. No clear between-group differences were observed for reading, transport-related sitting or other activities.

**Table 3:** Descriptive comparison of sedentary activity composition between high- and low-screen tendency groups.

Item	Item content	*M(IQR)*		*U* value	*Z* value	*P-*value
		High screen tendency group (*n* = 165)	Low screen tendency group (*n* = 114)			
SBQ1	Watching television, videos or DVDs	2.00 (0.29–3.00)	1.00 (0.00–2.00)	6533.00	−4.40	<0.001
SBQ2	Using a computer or the internet (e.g., tablets, smartphones or video games)	3.00 (1.00–4.00)	0.00 (0.00–2.00)	4466.50	−7.64	<0.001
SBQ3	Reading books, newspapers or magazines	0.00 (0.00–0.00)	0.00 (0.00–0.00)	9304.00	−0.29	0.773
SBQ4	Sitting and chatting or talking with relatives or friends	0.05 (0.00–0.33)	0.33 (0.00–1.00)	6616.50	−4.34	<0.001
SBQ5	Driving or riding in various means of transport	0.00 (0.00–0.14)	0.00 (0.00–0.19)	9362.00	−0.08	0.938
SBQ6	Eating meals (e.g., meals, snacks and tea or other beverages)	1.33 (1.00–1.50)	1.50 (0.00–2.00)	7579.00	−2.83	0.005
SBQ7	Hobbies (listening to music or radio, handicrafts, playing cards or chess)	0.43 (0.00–1.50)	2.00 (0.43–3.00)	5937.00	−5.33	<0.001
SBQ8	Napping (dozing while sitting or taking an afternoon nap)	1.00 (0.50–2.00)	1.25 (1.00–2.00)	7162.00	−3.43	0.001
SBQ9	Seated work or voluntary work	0.00 (0.00–0.00)	0.00 (0.00–0.50)	7877.50	−3.06	0.002
SBQ10	Other activities (e.g., attending church or temples)	0.00 (0.00–0.29)	0.00 (0.00–0.14)	8614.00	−1.45	0.147

SBQ1 and SBQ2 were components of the screen-based sedentary proportion used for group classification; their between-group comparisons should therefore be interpreted in relation to the operational group definition.

### 3.5 Multivariable logistic regression analysis of sedentary behavior

The results of the two-stage sequential models are presented in Table 4. High sedentary time status was related to physical function, living context, neighborhood infrastructure and exercise-related cognition. Participants with subjective lower-limb weakness had increased odds of high sedentary time status (OR 2.018, 95% CI: 1.098–3.711). Unmarried status and residence in an urban area were also linked to a higher likelihood of a high sedentary time status. In particular, participants residing in urban areas showed greater odds of high sedentary time status compared with those residing in rural areas (OR 3.461, 95% CI: 1.712–7.000). Higher scores for neighborhood infrastructure and outcome expectations for exercise were linked to reduced odds of high sedentary time status. The exercising alone dimension was linked to increased odds of high sedentary time status, whereas resistance from other dimensions was linked to reduced odds. The Nagelkerke R^2^ for Model I was 0.383.

**Table 4:** Factors associated with high sedentary time status and screen-based content differentiation according to multivariable logistic regression models.

Theoretical domain	Variable	Model I (*n* = 419)		Model II (*n* = 279)	
		*P-*value	OR (95% CI)	*P-*value	OR (95% CI)
Personal factors	Age (years)	—	—	0.034	0.950 (0.906–0.996)
	2hPG	—	—	0.039	1.066 (1.003–1.133)
	6-m gait speed (Reference = Higher performance)	—	—	0.130	—
	Lower performance vs. Higher performance	—	—	0.044	0.427 (0.187–0.976)
	Subjective lower-limb weakness				
	Yes vs. No	0.024	2.018 (1.098–3.711)	—	—
	Marital status				
	Unmarried vs. Married	0.035	2.762 (1.074–7.098)	—	—
	Residence				
	Urban vs. Rural	0.001	3.461 (1.712–7.000)	—	—
	Living arrangement (Reference = With spouse)	—	—	0.006	—
	With children vs. With spouse	—	—	0.005	0.286 (0.120–0.683)
Environmental factors	Neighborhood infrastructure	0.001	0.548 (0.379–0.793)	—	—
Behavioral cognitive factors	OEES	0.001	0.543 (0.377–0.782)	—	—
	Exercising alone	0.004	1.254 (1.076–1.462)	—	—
	Resistance from others	0.002	0.803 (0.701–0.920)	—	—
Model fit		R^2^ = 0.383, χ^2^ = 135.404, *P* < 0.001		R^2^ = 0.218, χ^2^ = 49.102, *P* < 0.001	

OR denotes odds ratio; 95% CI denotes 95% confidence interval. “—” indicates that the coefficient was not reported in the corresponding model because it was nonsignificant or not applicable. Outcome coding: high sedentary time group = 1, low sedentary time group = 0; high screen tendency = 1, low screen tendency = 0.

Among participants with high sedentary time, younger participants and higher 2hPG were linked to higher screen tendencies. Although the overall test for 6-m GS was not statistically significant (*p* = 0.130), the contrast between lower and higher performance showed a category-specific association (OR 0.427, 95% CI 0.187–0.976). This finding should be interpreted cautiously. Participants living with children had a lower likelihood of showing high screen tendencies (OR 0.286, 95% CI: 0.120–0.683). The Nagelkerke *R*^2^ for Model II was 0.218. In the complete-case sensitivity analysis after excluding participants with missing 2hPG or HbA1c values, the main findings of Model II remained broadly consistent with the primary analysis (Supplementary Appendix 3). Overall, high sedentary time status and sedentary content differentiation were associated with different factors. Table 4 presents the principal model results, and the full coefficients are reported in Supplementary Appendix 4.

### 3.6 Continuous outcome sensitivity analyses

To assess robustness to the >6 h/day and 0.35 operational cut-offs, three continuous-outcome sensitivity analyses were conducted: total sedentary time in the full sample (*n* = 419), the screen-based sedentary proportion among participants with high sedentary time (*n* = 279), and the screen-based sedentary proportion in the full sample (*n* = 419). All three models were statistically significant. The continuous total sedentary time analysis supported several principal associations identified in Model I. In the high-sedentary-time subgroup, living arrangement was significantly associated with the continuous screen-based sedentary proportion. The overall test for gait-speed performance was not statistically significant, although the contrast between lower and higher performance was significant. In the full-sample analysis, significant associations included 2hPG, gait speed and living arrangement. Full results are provided in Supplementary Appendices 5–7.

## 4 Discussion

In the present multicenter T2DM sample, poor metabolic control and functional limitations were both common. High sedentary time was also common in this sample, with 66.6% of participants classified as having high sedentary time, a proportion similar to that reported in recent studies of comparable populations (7). In addition, clear content heterogeneity was observed among participants with high sedentary time, with 59.1% classified as having high screen tendency. These findings indicated that sedentary behavior might be characterized not merely by accumulated time but also by differences in sedentary content. Further analysis showed that high sedentary time status was more strongly related to physical function, environmental support and exercise-related cognition, whereas sedentary content was more closely characterized by age, metabolic status and living arrangement, with only a category-specific contrast observed for gait speed. The continuous-outcome sensitivity analyses broadly supported these overall patterns, although some individual associations varied across analytical specifications. This finding suggests that future health promotion strategies should consider both sedentary time and sedentary content. Given the cross-sectional design, the direction of these associations cannot be determined, particularly for physical function and metabolic parameters.

Sedentary behavior involves not only total duration but also whether prolonged sitting can be interrupted. Previous sedentary behavior intervention studies in older adults have also treated sit-to-stand transitions and sedentary breaks as important outcomes (44). In this study, subjective lower-limb weakness remained independently associated with high sedentary time status, with odds approximately 2.02 times those of participants without subjective lower-limb weakness. A meta-analysis also showed that functional limitations often coexist with a higher sedentary behavior burden in older adults with diabetes (45). Reduced muscle strength does not necessarily mean complete inability to move, but it may make the transition from sitting to standing or walking more difficult (10). For older patients, this additional physical burden may make short periods of standing up more difficult (46). Therefore, sedentary behavior assessment and intervention research may extend beyond health education to consider lower-limb strength and sit-to-stand ability.

However, beyond physical function, external living contexts were also associated with high sedentary time status (3). In this study, urban residence was associated with higher odds of high sedentary time status than rural residence. This finding does not simply indicate that urban environments are less supportive of activity. Urban–rural differences in sedentary behavior may reflect multiple factors, including transport modes, public space, population density and lifestyle (47). Concurrently, greater neighborhood infrastructure support was associated with lower odds of high sedentary time status. Accessible walking routes, resting facilities and public spaces may reduce the environmental cost of interrupting sitting and engaging in short bouts of activity (48). This finding is consistent with the environmental perspective of SCT, which emphasizes the role of external conditions in shaping behavior. When walking routes and public spaces are more supportive, standing up and short periods of activity may be more easily incorporated into daily life (49). Health advice may move beyond general encouragement to be active by helping patients identify accessible activity places and incorporate standing reminders into fixed daily routines.

Physical function and living context may provide conditions for behavior to occur, while exercise-related cognition may influence whether these conditions are translated into daily action (23). In this study, outcome expectations for exercise were a prominent cognitive factor. Patients reporting more favorable expectations had a lower likelihood of being sedentary, which is consistent with TPB, where positive behavioral beliefs support favorable attitudes and behavioral intention (24). The self-efficacy findings were more nuanced. The exercising alone dimension was positively associated with high sedentary time status, whereas the resistance from others dimension was negatively associated with high sedentary time status. This finding suggests that higher self-efficacy should not be interpreted uniformly across dimensions (36). Confidence in exercising alone may mainly reflect perceived ability to complete planned exercise, whereas reducing sedentary behavior requires repeated standing, interruption of prolonged sitting and adjustment across daily contexts (50, 51). In contrast, confidence in dealing with resistance from others may better reflect the ability to maintain activity during social interactions (52). Behavioral interventions could embed standing-up activities into specific times, contexts and barriers, rather than focusing only on strengthening confidence in general.

Among participants with high sedentary time, sedentary content differentiation showed a pattern of associated factors that differed from those related to high sedentary time status. A higher proportion of screen-based sedentary time was more common among participants with high sedentary time who were younger, had higher 2hPG. Although the contrast between lower and higher gait-speed performance showed a category-specific association, the overall gait-speed variable was not statistically significant. This observation suggests that sedentary content is not simply an extension of total sedentary volume, but rather reflects how individuals use time in specific contexts (53). Hudgins also noted that distinguishing screen-related from non-screen-related sedentary behavior can reveal more specific behavioral differences than total sedentary time alone (54). Similar differences were observed within the sedentary subgroup in the present study, further suggesting that high screen tendency may have relatively distinct associated characteristics (55). Younger older adults may have easier access to mobile phones, computers and tablets, and their sedentary time may therefore be more concentrated in screen-based activities (55). This conjecture is consistent with evidence from a large Brazilian study, in which device-related sedentary behavior was more prominent in younger groups (56). The positive association between 2hPG and high screen tendency should be interpreted with caution, as the cross-sectional design cannot determine directionality. However, previous research has linked sedentary time with postprandial glucose responses and glycaemic control (57). Therefore, examining high screen tendency during the postprandial period may be closer to a real-life context for future longitudinal and intervention studies than focusing only on reducing total sedentary time.

The item-level analysis further described the activity composition of high screen tendency. High screen tendency was accompanied by more screen-based sedentary duration and less duration in several nonscreen sedentary activities including chatting, hobbies, meals and napping. These differences were selective, as reading, transport-related sitting and other activities did not differ significantly between the groups. This observation suggests that high screen tendency may reflect differences in time allocation across sedentary activities, rather than simply longer sedentary time. However, these findings do not establish that screen-based activities directly displaced nonscreen activities. This distinction is important because some seated activities in older adults may have social, cognitive or restorative value (58). In contrast, screen-based sedentary behavior may have different psychological and behavioral characteristics compared with non-screen-based sedentary behaviors (54).

Living arrangement also appeared relevant. Participants living with children were less likely to show a high screen tendency, which may be related to family interaction and daily activity arrangements (59). In China, older adults living with adult children often participate in the care of grandchildren and intergenerational support. These family responsibilities may embed their daily time more in caregiving, companionship and transport-related activities, thereby reducing opportunities for prolonged screen exposure (60). These findings suggest that sedentary behavior management for older inpatients with T2DM may need to consider not only the amount of sedentary time but also the activities performed while sitting. In particular, future interventions could aim to reduce prolonged continuous screen exposure while considering the potential value of appropriate social, hobby-related or restorative sedentary activities.

## 5 Strengths and limitations

In this research, we primarily examined sedentary content in addition to total sedentary time, and further analyzed the associated characteristics of high screen tendency among participants with high sedentary time. This two-stage sequential analysis helped reduce overlap between factors related to different stages of sedentary behavior. Second, this study combined clinical indicators, field-based physical measurements and questionnaire data, allowing functional status, environmental support and exercise-related cognition to be examined within the same framework. The item-level analysis of sedentary activities also provided a more complete description of the structure of sedentary behavior. In addition, the multicenter sample included tertiary hospitals from different regions of China, which broadened the sample source and reflected real-world clinical contexts.

Several limitations related to this study should be noted. First, the cross-sectional design precludes causal inference. Second, sedentary behavior was mainly assessed using a self-reported questionnaire and may therefore be affected by recall bias. Although this study further analyzed specific sedentary activity items, it did not use objective devices to monitor prolonged sedentary bouts or sedentary breaks. In addition, information on diabetic complications, multimorbidity and depressive symptoms was not systematically collected, and residual confounding cannot be excluded. Associations involving physical function should therefore be interpreted cautiously. Participants with cognitive impairment or mental disorders were also excluded because the study required independent communication and questionnaire completion, which may limit the applicability of the findings to older adults with more complex clinical conditions. In addition, participants were recruited from tertiary hospitals and were older inpatients with T2DM, although sedentary behavior information was based mainly on recall of the home setting prior to admission. This sample helps to describe daily sedentary characteristics among older patients in clinical care, but its applicability to the wider older T2DM population requires further validation. Potential differences in patient characteristics and clinical settings across the three centers should also be considered when interpreting the pooled findings. Although the continuous-outcome sensitivity analyses reduced concern that the principal findings were solely driven by the operational cut-offs, they do not establish clinically validated thresholds. Future studies could combine longitudinal designs, objective device-based monitoring, and clinical, outpatient or community settings to further evaluate lifestyle intervention strategies targeting high sedentary time status and high screen tendency. External validation of these classifications and compositional approaches are also needed to further examine how sedentary time is distributed across activity types.

## 6 Conclusion

Overall, there was clear heterogeneity in the sedentary behavior of older adults with T2DM. The characteristics of high sedentary time status and sedentary content differentiation were associated with different factors. Compared with focusing only on total sedentary time, further identifying high screen tendency and the accompanying distribution of daily sedentary activities could better reflect the real-life contexts of older patients. The continuous-outcome sensitivity analyses broadly supported this distinction, although some individual associations varied across model specifications. For public health practice, sedentary behavior management may need to go beyond reducing sitting time and also consider what patients do while sitting, and how these activities are embedded in their functional status, family context and daily life. The relevance of these potential targets requires confirmation in longitudinal and interventional studies.

## Data Availability

The original contributions presented in the study are included in the article/Supplementary material, further inquiries can be directed to the corresponding author/s.
