# Supplementary material for: From sedentary volume to sedentary content in older inpatients with type 2 diabetes: a multicenter study
Source: Front Public Health. 2026 Aug 20;14:1907446. doi: 10.3389/fpubh.2026.1907446 (PMC13520833; doi:10.3389/fpubh.2026.1907446)
Supplement: Supplementary file 1 [file Supplementary_file_1.docx]

**From sedentary volume to sedentary content in older inpatients with type 2 diabetes: A multicentre study**

**Supplementary Data (Appendices 1–7)**

**Contents list**

**Appendix 1**. Intraclass correlation coefficient (ICC) for the SBQ

**Appendix 2**. Full descriptive characteristics and univariate comparisons for high sedentary time status and screen-based content differentiation

**Appendix 3**. Complete-case sensitivity analysis for Model II after excluding participants with missing clinical indicators

**Appendix 4**. Full coefficients for high sedentary time status and screen-based content differentiation models

**Appendix 5**. Continuous-outcome sensitivity analysis for Model I using total sedentary time

**Appendix 6**. Continuous screen-based sedentary proportion analysis among participants with high sedentary time

**Appendix 7**. Additional full-sample continuous screen-based sedentary proportion analysis

**Appendix 1.** Intraclass correlation coefficient (ICC) for the SBQ

| **Scale item** | ***ICC*** | ***95% CI* (bootstrap)** | | ***P value*** |
| --- | --- | --- | --- | --- |
|  |  | **lower** | **upper** |  |
| SBQ1 | 0.751 | 0.576 | 0.861 | <0.001 |
| SBQ2 | 0.942 | 0.861 | 0.973 | <0.001 |
| SBQ3 | 0.689 | 0.485 | 0.822 | <0.001 |
| SBQ4 | 0.743 | 0.564 | 0.856 | <0.001 |
| SBQ5 | 0.484 | 0.205 | 0.689 | 0.001 |
| SBQ6 | 0.720 | 0.529 | 0.842 | <0.001 |
| SBQ7 | 0.841 | 0.720 | 0.912 | <0.001 |
| SBQ8 | 0.726 | 0.541 | 0.845 | <0.001 |
| SBQ9 | 0.948 | 0.904 | 0.972 | <0.001 |
| SBQ10 | 0.771 | 0.608 | 0.872 | <0.001 |
| Total SBQ | 0.881 | 0.785 | 0.936 | <0.001 |

**Appendix 2.** Full descriptive characteristics and univariate comparisons for high sedentary time status and screen-based content differentiation

| **Variable** | **High sedentary time group**  **(n = 279)** | **Low sedentary time group**  **(n = 140)** | **t/χ²** | ***P value*** | **High screen tendency group (n = 165)** | **Low screen tendency group (n = 114)** | **t/χ²** | ***P value*** |
| --- | --- | --- | --- | --- | --- | --- | --- | --- |
| Age (years) | 69.81±8.26 | 69.26±6.26 | 0.752 | 0.453 | 68.41±7.28 | 71.83±9.12 | -3.342 | 0.001 |
| BMI (kg/m²) | 24.86±4.05 | 24.59±3.66 | 0.652 | 0.515 | 24.83±4.26 | 24.90±3.74 | -0.152 | 0.879 |
| 2hPG | 15.64±5.19 | 14.31±6.17 | 2.187 | 0.030 | 16.22±5.01 | 14.80±5.35 | 2.262 | 0.024 |
| HbA1c (%) | 8.73±2.09 | 9.22±2.52 | -2.088 | 0.037 | 8.73±2.09 | 8.73±2.10 | 0.013 | 0.990 |
| **SSRS** |  |  |  |  |  |  |  |  |
| Subjective support | 17.89±4.74 | 19.74±4.17 | -4.080 | ＜0.001 | 18.14±4.67 | 17.53±4.84 | 1.062 | 0.289 |
| Objective support | 8.72±2.54 | 9.16±2.63 | -1.653 | 0.099 | 8.80±2.66 | 8.61±2.37 | 0.600 | 0.549 |
| Support utilisation | 6.61±2.00 | 6.78±2.18 | -0.809 | 0.419 | 6.58±1.99 | 6.65±2.04 | -0.300 | 0.764 |
| **PANES** |  |  |  |  |  |  |  |  |
| Destination accessibility | 3.41±0.60 | 3.46±0.74 | -0.795 | 0.427 | 3.43±0.60 | 3.37±0.59 | 0.775 | 0.439 |
| Neighbourhood infrastructure | 2.95±0.92 | 3.55±0.69 | -7.391 | ＜0.001 | 3.03±0.91 | 2.85±0.94 | 1.605 | 0.110 |
| Social environment | 3.26±0.87 | 3.61±0.73 | -4.329 | 0.001 | 3.24±0.87 | 3.29±0.88 | -0.411 | 0.682 |
| Aesthetic value | 3.20±0.93 | 3.59±0.76 | -4.600 | ＜0.001 | 3.17±0.95 | 3.24±0.89 | -0.633 | 0.528 |
| Street connectivity | 3.03±0.96 | 3.42±0.81 | -4.174 | ＜0.001 | 3.04±1.00 | 3.02±0.89 | 0.165 | 0.869 |
| Neighbourhood safety | 3.10±0.62 | 3.34±0.56 | -3.892 | ＜0.001 | 3.14±0.60 | 3.04±0.63 | 1.238 | 0.204 |
| Residential density | 4.07±1.23 | 3.58±1.43 | 3.499 | 0.001 | 4.21±1.12 | 3.87±1.34 | 2.238 | 0.026 |
| Household motor vehicle ownership | 0.51±0.71 | 0.87±0.88 | -4.232 | ＜0.001 | 0.47±0.65 | 0.56±0.79 | -0.992 | 0.322 |
| **OEES** | 3.43±0.92 | 4.04±0.79 | -7.072 | ＜0.001 | 3.44±0.91 | 3.41±0.94 | 0.280 | 0.780 |
| **ESE** |  |  |  |  |  |  |  |  |
| Negative affect | 8.54±4.21 | 10.65±4.18 | -4.840 | ＜0.001 | 8.70±4.32 | 8.32±4.06 | 0.726 | 0.469 |
| Making excuses | 7.30±4.22 | 9.40±4.68 | -4.474 | ＜0.001 | 7.64±4.37 | 6.82±3.96 | 1.602 | 0.110 |
| Exercising alone | 9.95±3.73 | 11.43±3.49 | -3.919 | ＜0.001 | 10.16±3.78 | 9.63±3.65 | 1.172 | 0.242 |
| Lack of exercise equipment | 9.69±4.08 | 11.91±3.27 | -6.010 | ＜0.001 | 10.13±4.02 | 9.06±4.10 | 2.160 | 0.032 |
| Resistance from others | 8.24±4.27 | 11.09±3.71 | -7.046 | ＜0.001 | 8.39±4.30 | 8.04±4.24 | 0.678 | 0.499 |
| Bad weather | 6.05±3.81 | 7.13±4.42 | -2.470 | 0.014 | 6.10±3.80 | 5.97±3.85 | 0.265 | 0.791 |
| Handgrip strength | 22.78±9.20 | 23.08±8.68 | -0.325 | 0.745 | 23.22±9.77 | 22.14±8.30 | 0.966 | 0.335 |
| **Five-times-sit-to-stand test (5TCS)** |  |  | 8.960 | 0.011 |  |  | 5.525 | 0.063 |
| Higher performance | 104 | 70 |  |  | 69 | 35 |  |  |
| Lower performance | 118 | 55 |  |  | 69 | 49 |  |  |
| Uncompleted | 57 | 15 |  |  | 27 | 30 |  |  |
| **6-m gait speed (6-m GS)** |  |  | 4.448 | 0.108 |  |  | 8.990 | 0.011 |
| Higher performance | 110 | 66 |  |  | 77 | 33 |  |  |
| Lower performance | 119 | 59 |  |  | 63 | 56 |  |  |
| Uncompleted | 50 | 15 |  |  | 25 | 25 |  |  |
| **Subjective lower-limb weakness** |  |  | 19.498 | ＜0.001 |  |  | 1.842 | 0.175 |
| Yes | 143 | 40 |  |  | 79 | 64 |  |  |
| No | 136 | 100 |  |  | 86 | 50 |  |  |
| **Gender** |  |  | 1.300 | 0.254 |  |  | 0.139 | 0.709 |
| Male | 148 | 66 |  |  | 86 | 62 |  |  |
| Female | 131 | 74 |  |  | 79 | 52 |  |  |
| **Family history of diabetes** |  |  | 6.455 | 0.011 |  |  | 0.922 | 0.337 |
| Yes | 132 | 48 |  |  | 82 | 50 |  |  |
| No | 147 | 92 |  |  | 83 | 64 |  |  |
| **Marital status** |  |  | 6.408 | 0.011 |  |  | 3.143 | 0.076 |
| Unmarried | 84 | 26 |  |  | 43 | 41 |  |  |
| Married | 195 | 114 |  |  | 122 | 73 |  |  |
| **Monthly household income (CNY)** |  |  | 1.554 | 0.213 |  |  | 6.700 | 0.010 |
| < 2000 | 66 | 41 |  |  | 30 | 36 |  |  |
| ≥ 2000 | 213 | 99 |  |  | 135 | 78 |  |  |
| **Education level** |  |  | 5.446 | 0.066 |  |  | 6.552 | 0.038 |
| Primary school | 58 | 42 |  |  | 26 | 32 |  |  |
| Junior high school | 94 | 48 |  |  | 57 | 37 |  |  |
| Senior high school or above | 127 | 50 |  |  | 82 | 45 |  |  |
| **Living environment** |  |  | 22.947 | ＜0.001 |  |  | 9.051 | 0.003 |
| Rural | 38 | 47 |  |  | 14 | 24 |  |  |
| Urban | 241 | 93 |  |  | 151 | 90 |  |  |
| **Living arrangement** |  |  | 2.384 | 0.304 |  |  | 16.432 | < 0.001 |
| With spouse | 158 | 84 |  |  | 105 | 53 |  |  |
| With children | 85 | 45 |  |  | 35 | 50 |  |  |
| Alone or with others | 36 | 11 |  |  | 25 | 11 |  |  |

**Appendix 3.** Complete-case sensitivity analysis for Model II (n=265) after excluding participants with missing clinical indicators

|  | **Theoretical domain** | | **Variable** | | ***B*** | ***SE*** | | | ***Wald*** | | ***P value*** | ***OR*** | | ***95% CI*** | | | |
| --- | --- | --- | --- | --- | --- | --- | --- | --- | --- | --- | --- | --- | --- | --- | --- | --- | --- |
|  | Personal factors | | Age, years | | -0.058 | 0.026 | | | 5.125 | | 0.024 | 0.943 | | 0.897 | | 0.992 | |
|  |  |  | BMI | | 0.028 | 0.036 | | | 0.606 | | 0.436 | 1.029 | | 0.958 | | 1.104 | |
|  |  |  | 2hPG | | 0.067 | 0.033 | | | 4.249 | | 0.039 | 1.070 | | 1.003 | | 1.141 | |
|  |  |  | HbA1c | | -0.098 | 0.081 | | | 1.481 | | 0.224 | 0.906 | | 0.773 | | 1.062 | |
|  |  |  | Handgrip strength | | -0.042 | 0.024 | | | 2.989 | | 0.084 | 0.959 | | 0.915 | | 1.006 | |
|  |  |  | **Five-times-sit-to-stand test (Reference = Higher performance)** | |  |  | | |  | |  |  | |  | |  | |
|  |  |  | Lower performance vs Higher performance | | 0.209 | 0.409 | | | 0.261 | | 0.610 | 1.232 | | 0.553 | | 2.749 | |
|  |  |  | Uncompleted vs Higher performance | | 0.491 | 0.733 | | | 0.450 | | 0.503 | 1.634 | | 0.389 | | 6.870 | |
|  |  |  | **6-m gait speed (Reference = Higher performance)** | |  |  | | |  | |  |  | |  | |  | |
|  |  |  | Lower performance vs Higher performance | | -0.806 | 0.432 | | | 3.480 | | 0.062 | 0.447 | | 0.191 | | 1.042 | |
|  |  |  | Uncompleted vs Higher performance | | -1.200 | 0.726 | | | 2.731 | | 0.098 | 0.301 | | 0.073 | | 1.250 | |
|  |  |  | **Subjective lower-limb weakness** | |  |  | | |  | |  |  | |  | |  | |
|  |  |  | Yes vs No | | 0.461 | 0.361 | | | 1.626 | | 0.202 | 1.585 | | 0.781 | | 3.217 | |
|  |  |  | **Gender** | |  |  | | |  | |  |  | |  | |  | |
|  |  |  | Male vs Female | | -0.051 | 0.351 | | | 0.021 | | 0.884 | 0.950 | | 0.477 | | 1.892 | |
|  |  |  | **Family history of diabetes** | |  |  | | |  | |  |  | |  | |  | |
|  |  |  | Yes vs No | | 0.220 | 0.286 | | | 0.591 | | 0.442 | 1.246 | | 0.711 | | 2.184 | |
|  |  |  | **Marital status** | |  |  | | |  | |  |  | |  | |  | |
|  |  |  | Unmarried vs Married | | 0.322 | 0.519 | | | 0.384 | | 0.535 | 1.379 | | 0.499 | | 3.815 | |
|  |  |  | **Monthly household income, CNY** | |  |  | | |  | |  |  | |  | |  | |
|  |  |  | ≥2000 vs <2000 | | 0.171 | 0.418 | | | 0.167 | | 0.682 | 1.186 | | 0.523 | | 2.690 | |
|  |  |  | **Education level (Reference = Primary school)** | |  |  | | |  | |  |  | |  | |  | |
|  |  |  | Junior high school vs Primary school | | 0.412 | 0.453 | | | 0.825 | | 0.364 | 1.509 | | 0.621 | | 3.669 | |
|  |  |  | Senior high school or above vs Primary school | | 0.125 | 0.471 | | | 0.070 | | 0.791 | 1.133 | | 0.450 | | 2.850 | |
|  |  |  | **Residence** | |  |  | | |  | |  |  | |  | |  | |
|  |  |  | Urban vs Rural | | 0.896 | 0.597 | | | 2.257 | | 0.133 | 2.450 | | 0.761 | | 7.888 | |
|  |  |  | **Living arrangement (Reference = With spouse)** | |  |  | | |  | |  |  | |  | |  | |
|  |  |  | With children vs With spouse | | -1.300 | 0.448 | | | 8.408 | | 0.004 | 0.273 | | 0.113 | | 0.656 | |
|  |  |  | Alone or with others vs With spouse | | -0.209 | 0.630 | | | 0.110 | | 0.740 | 0.812 | | 0.236 | | 2.789 | |
| Environmental factors | | Residential density | | 0.136 | | | 0.146 | 0.866 | | 0.352 | | 1.145 | 0.860 | | 1.525 | |  |
| Behavioural cognitive factors | | Lack of exercise equipment | | 0.030 | | | 0.044 | 0.458 | | 0.499 | | 1.030 | 0.945 | | 1.124 | |  |
| Model Fit | | Nagelkerke R² = 0.234; χ² = 50.471; P <0.001 | | | | | | | | | | | | | | |  |

**Notes.** The complete-case sensitivity analysis excluded 1 participant with missing 2hPG and 13 participants with missing HbA1c values, resulting in a final sample of 265 participants.

**Appendix 4.** Full coefficients for high sedentary time status and screen-based content differentiation models

**Model I (n=419)**

| **Theoretical domain** | **Variable** | ***B*** | ***SE*** | ***Wald*** | ***P value*** | ***OR*** | ***95% CI*** | |
| --- | --- | --- | --- | --- | --- | --- | --- | --- |
| Personal factors | Age, years | -0.032 | 0.022 | 2.128 | 0.145 | 0.969 | 0.928 | 1.011 |
|  | BMI | 0.066 | 0.034 | 3.685 | 0.055 | 1.068 | 0.999 | 1.142 |
|  | 2hPG | 0.031 | 0.026 | 1.508 | 0.219 | 1.032 | 0.981 | 1.085 |
|  | HbA1c | -0.043 | 0.062 | 0.476 | 0.490 | 0.958 | 0.848 | 1.082 |
|  | Handgrip strength | -0.024 | 0.023 | 1.169 | 0.280 | 0.976 | 0.934 | 1.020 |
|  | **Five-times-sit-to-stand test (Reference = Higher performance)** |  |  | 0.076 | 0.963 |  |  |  |
|  | Lower performance vs Higher performance | 0.053 | 0.306 | 0.030 | 0.863 | 1.054 | 0.579 | 1.920 |
|  | Uncompleted vs Higher performance | 0.148 | 0.567 | 0.069 | 0.793 | 1.160 | 0.382 | 3.524 |
|  | **6-m gait speed (Reference = Higher performance)** |  |  | 3.623 | 0.163 |  |  |  |
|  | Lower performance vs Higher performance | -0.615 | 0.325 | 3.572 | 0.059 | 0.541 | 0.286 | 1.023 |
|  | Uncompleted vs Higher performance | -0.247 | 0.557 | 0.196 | 0.658 | 0.781 | 0.262 | 2.328 |
|  | **Subjective lower-limb weakness** |  |  |  |  |  |  |  |
|  | Yes vs No | 0.702 | 0.311 | 5.111 | 0.024 | 2.018 | 1.098 | 3.711 |
|  | **Gender** |  |  |  |  |  |  |  |
|  | Male vs Female | 0.609 | 0.346 | 3.096 | 0.078 | 1.839 | 0.933 | 3.626 |
|  | **Family history of diabetes** |  |  |  |  |  |  |  |
|  | Yes vs No | 0.509 | 0.261 | 3.797 | 0.051 | 1.664 | 0.997 | 2.777 |
|  | **Marital status** |  |  |  |  |  |  |  |
|  | Unmarried vs Married | 1.016 | 0.482 | 4.448 | 0.035 | 2.762 | 1.074 | 7.098 |
|  | **Monthly household income, CNY** |  |  |  |  |  |  |  |
|  | ≥2000 vs <2000 | 0.259 | 0.379 | 0.468 | 0.494 | 1.296 | 0.616 | 2.725 |
|  | **Education level (Reference = Primary school)** |  |  | 3.486 | 0.175 |  |  |  |
|  | Junior high school vs Primary school | 0.672 | 0.363 | 3.430 | 0.064 | 1.959 | 0.962 | 3.990 |
|  | Senior high school or above vs Primary school | 0.565 | 0.397 | 2.024 | 0.155 | 1.760 | 0.808 | 3.836 |
|  | **Residence** |  |  |  |  |  |  |  |
|  | Urban vs Rural | 1.242 | 0.359 | 11.943 | 0.001 | 3.461 | 1.712 | 7.000 |
|  | **Living arrangement (Reference = With spouse)** |  |  | 0.889 | 0.641 |  |  |  |
|  | With children vs With spouse | -0.320 | 0.352 | 0.824 | 0.364 | 0.726 | 0.364 | 1.449 |
|  | Alone or with others vs With spouse | -0.406 | 0.608 | 0.445 | 0.505 | 0.666 | 0.202 | 2.194 |
| Environmental factors | Neighbourhood infrastructure | -0.601 | 0.188 | 10.201 | 0.001 | 0.548 | 0.379 | 0.793 |
| Behavioural cognitive factors | **OEES** | -0.611 | 0.186 | 10.750 | 0.001 | 0.543 | 0.377 | 0.782 |
|  | Exercising alone | 0.226 | 0.078 | 8.372 | 0.004 | 1.254 | 1.076 | 1.462 |
|  | Resistance from others | -0.219 | 0.069 | 9.984 | 0.002 | 0.803 | 0.701 | 0.920 |
| Model fit | Nagelkerke R^2^=0.383，χ^2^=135.404,*P*<0.001 | | | | | | | |

**ModelII (n=279)**

| **Theoretical domain** | **Variable** | ***B*** | ***SE*** | ***Wald*** | ***P value*** | ***OR*** | ***95% CI*** | |
| --- | --- | --- | --- | --- | --- | --- | --- | --- |
| Personal factors | Age, years | -0.052 | 0.024 | 4.515 | 0.034 | 0.950 | 0.906 | 0.996 |
|  | BMI | 0.033 | 0.035 | 0.921 | 0.337 | 1.034 | 0.966 | 1.107 |
|  | 2hPG | 0.064 | 0.031 | 4.273 | 0.039 | 1.066 | 1.003 | 1.133 |
|  | HbA1c | -0.100 | 0.079 | 1.594 | 0.207 | 0.905 | 0.776 | 1.057 |
|  | Handgrip strength | -0.044 | 0.023 | 3.475 | 0.062 | 0.957 | 0.914 | 1.002 |
|  | **Five-times-sit-to-stand test (Reference = Higher performance)** |  |  | 0.473 | 0.789 |  |  |  |
|  | Lower performance vs Higher performance | 0.105 | 0.399 | 0.070 | 0.792 | 1.111 | 0.508 | 2.431 |
|  | Uncompleted vs Higher performance | -0.313 | 0.673 | 0.216 | 0.642 | 0.731 | 0.196 | 2.734 |
|  | **6-m gait speed (Reference = Higher performance)** |  |  | 4.082 | 0.130 |  |  |  |
|  | Lower performance vs Higher performance | -0.850 | 0.421 | 4.070 | 0.044 | 0.427 | 0.187 | 0.976 |
|  | Uncompleted vs Higher performance | -0.581 | 0.688 | 0.715 | 0.398 | 0.559 | 0.145 | 2.152 |
|  | **Subjective lower-limb weakness** |  |  |  |  |  |  |  |
|  | Yes vs No | 0.354 | 0.349 | 1.024 | 0.312 | 1.424 | 0.718 | 2.825 |
|  | **Gender** |  |  |  |  |  |  |  |
|  | Male vs Female | -0.052 | 0.339 | 0.023 | 0.878 | 0.949 | 0.488 | 1.846 |
|  | **Family history of diabetes** |  |  |  |  |  |  |  |
|  | Yes vs No | 0.258 | 0.275 | 0.880 | 0.348 | 1.294 | 0.755 | 2.216 |
|  | **Marital status** |  |  |  |  |  |  |  |
|  | Unmarried vs Married | 0.553 | 0.509 | 1.180 | 0.277 | 1.739 | 0.641 | 4.719 |
|  | **Monthly household income, CNY** |  |  |  |  |  |  |  |
|  | ≥2000 vs <2000 | 0.244 | 0.398 | 0.375 | 0.540 | 1.276 | 0.585 | 2.783 |
|  | **Education level (Reference = Primary school)** |  |  | 1.799 | 0.407 |  |  |  |
|  | Junior high school vs Primary school | 0.468 | 0.421 | 1.237 | 0.266 | 1.597 | 0.700 | 3.647 |
|  | Senior high school or above vs Primary school | 0.088 | 0.440 | 0.040 | 0.842 | 1.092 | 0.461 | 2.588 |
|  | **Residence** |  |  |  |  |  |  |  |
|  | Urban vs Rural | 0.752 | 0.566 | 1.765 | 0.184 | 2.121 | 0.699 | 6.430 |
|  | **Living arrangement (Reference = With spouse)** |  |  | 10.230 | 0.006 |  |  |  |
|  | With children vs With spouse | -1.252 | 0.444 | 7.954 | 0.005 | 0.286 | 0.120 | 0.683 |
|  | Alone or with others vs With spouse | -0.294 | 0.620 | 0.225 | 0.635 | 0.745 | 0.221 | 2.510 |
| Environmental factors | Residential density | 0.147 | 0.142 | 1.069 | 0.301 | 1.158 | 0.877 | 1.529 |
| Behavioural cognitive factors | Lack of exercise equipment | 0.012 | 0.043 | 0.085 | 0.771 | 1.012 | 0.931 | 1.101 |
| Model fit | Nagelkerke R^2^=0.218，χ^2^=49.102, *P*＜0.001 | | | | | | | |

**Notes.** OR = odds ratio; 95% CI = 95% confidence interval. “—” indicates that the coefficient was not reported in the corresponding model because it was non-significant or not applicable. Outcome coding: high sedentary time group = 1, low sedentary time group= 0; high screen tendency = 1, low screen tendency = 0

**Appendix 5.** Continuous-outcome sensitivity analysis for Model I using total sedentary time

Model I (n=419)

| **Theoretical domain** | **Variable** | ***B*** | ***HC3 robust SE*** | ***P value*** | ***95% CI*** | | ***VIF*** |
| --- | --- | --- | --- | --- | --- | --- | --- |
| Personal factors | Age, years | -0.027 | 0.021 | 0.191 | -0.068 | 0.014 | 1.822 |
|  | BMI | 0.070 | 0.031 | 0.027 | 0.008 | 0.131 | 1.104 |
|  | 2hPG | 0.056 | 0.030 | 0.062 | -0.003 | 0.114 | 1.440 |
|  | HbA1c | -0.100 | 0.071 | 0.159 | -0.239 | 0.039 | 1.420 |
|  | Handgrip strength | 0.004 | 0.023 | 0.872 | -0.042 | 0.050 | 2.625 |
|  | **Five-times-sit-to-stand test (Reference = Higher performance)** |  |  | 0.578 |  |  |  |
|  | Lower performance vs Higher performance | 0.219 | 0.314 | 0.486 | -0.398 | 0.837 | 1.649 |
|  | Uncompleted vs Higher performance | -0.259 | 0.608 | 0.670 | -1.453 | 0.936 | 3.181 |
|  | **6-m gait speed (Reference = Higher performance)** |  |  | 0.073 |  |  |  |
|  | Lower performance vs Higher performance | -0.649 | 0.317 | 0.041 | -1.272 | -0.026 | 1.831 |
|  | Uncompleted vs Higher performance | 0.026 | 0.566 | 0.963 | -1.086 | 1.138 | 2.914 |
|  | **Subjective lower-limb weakness** |  |  |  |  |  |  |
|  | Yes vs No | 1.083 | 0.320 | <0.001 | 0.454 | 1.713 | 1.638 |
|  | **Gender** |  |  |  |  |  |  |
|  | Male vs Female | 0.198 | 0.332 | 0.551 | -0.455 | 0.851 | 1.779 |
|  | **Family history of diabetes** |  |  |  |  |  |  |
|  | Yes vs No | 0.304 | 0.254 | 0.232 | -0.195 | 0.803 | 1.048 |
|  | **Marital status** |  |  |  |  |  |  |
|  | Unmarried vs Married | 0.863 | 0.450 | 0.056 | -0.022 | 1.748 | 2.614 |
|  | **Monthly household income, CNY** |  |  |  |  |  |  |
|  | ≥2000 vs <2000 | 0.639 | 0.352 | 0.070 | -0.053 | 1.331 | 1.665 |
|  | **Education level (Reference = Primary school)** |  |  | 0.436 |  |  |  |
|  | Junior high school vs Primary school | 0.253 | 0.393 | 0.520 | -0.519 | 1.026 | 1.997 |
|  | Senior high school or above vs Primary school | 0.502 | 0.404 | 0.215 | -0.293 | 1.298 | 2.565 |
|  | **Residence** |  |  |  |  |  |  |
|  | Urban vs Rural | 1.084 | 0.361 | 0.003 | 0.375 | 1.793 | 1.440 |
|  | **Living arrangement (Reference = With spouse)** |  |  | 0.525 |  |  |  |
|  | With children vs With spouse | -0.443 | 0.391 | 0.257 | -1.211 | 0.325 | 1.920 |
|  | Alone or with others vs With spouse | -0.328 | 0.576 | 0.570 | -1.460 | 0.805 | 2.074 |
| Environmental factors | Neighbourhood infrastructure | -0.462 | 0.177 | 0.009 | -0.809 | -0.115 | 1.558 |
| Behavioural cognitive factors | OEES mean score | -0.466 | 0.216 | 0.032 | -0.891 | -0.041 | 1.916 |
|  | Exercising alone | 0.076 | 0.067 | 0.258 | -0.056 | 0.208 | 4.108 |
|  | Resistance from others | -0.106 | 0.056 | 0.060 | -0.216 | 0.004 | 4.428 |
| **Model fit** | R²=0.265, adjusted R²=0.222, F=6.193, P<0.001 | | | | | | |

**Notes.** B denotes unstandardised coefficients. HC3 robust standard errors and 95% confidence intervals are reported. For multicategory predictors, parent-row P values are from joint HC3 robust Wald tests, whereas category-row P values refer to individual contrasts with the reference category. F is the conventional model F statistic

**Appendix 6.** Continuous screen-based sedentary proportion analysis among participants with high sedentary time

Model II (n=279)

| **Theoretical domain** | **Variable** | ***β coefficient*** | ***HC3 robust SE*** | ***P value*** | ***95% CI*** | | ***VIF*** |
| --- | --- | --- | --- | --- | --- | --- | --- |
| Personal factors | Age, years | -0.013 | 0.009 | 0.143 | -0.029 | 0.004 | 2.133 |
|  | BMI | -0.003 | 0.013 | 0.833 | -0.028 | 0.022 | 1.109 |
|  | 2hPG | 0.005 | 0.013 | 0.704 | -0.020 | 0.029 | 1.358 |
|  | HbA1c | 0.002 | 0.030 | 0.951 | -0.056 | 0.060 | 1.349 |
|  | Handgrip strength | -0.008 | 0.010 | 0.439 | -0.028 | 0.012 | 2.488 |
|  | **Five-times-sit-to-stand test (Reference = Higher performance)** |  |  | 0.943 |  |  |  |
|  | Lower performance vs Higher performance | 0.034 | 0.130 | 0.796 | -0.221 | 0.288 | 2.092 |
|  | Uncompleted vs Higher performance | 0.088 | 0.287 | 0.758 | -0.474 | 0.651 | 4.140 |
|  | **6-m gait speed (Reference = Higher performance)** |  |  | 0.074 |  |  |  |
|  | Lower performance vs Higher performance | -0.294 | 0.129 | 0.022 | -0.546 | -0.042 | 2.352 |
|  | Uncompleted vs Higher performance | -0.229 | 0.272 | 0.399 | -0.762 | 0.303 | 3.886 |
|  | **Subjective lower-limb weakness** |  |  |  |  |  |  |
|  | Yes vs No | 0.129 | 0.136 | 0.340 | -0.137 | 0.395 | 1.691 |
|  | **Gender** |  |  |  |  |  |  |
|  | Male vs Female | -0.025 | 0.134 | 0.854 | -0.286 | 0.237 | 1.611 |
|  | **Family history of diabetes** |  |  |  |  |  |  |
|  | Yes vs No | 0.121 | 0.098 | 0.217 | -0.071 | 0.314 | 1.052 |
|  | **Marital status** |  |  |  |  |  |  |
|  | Unmarried vs Married | -0.002 | 0.223 | 0.994 | -0.440 | 0.436 | 3.177 |
|  | **Monthly household income, CNY** |  |  |  |  |  |  |
|  | ≥2000 vs <2000 | -0.037 | 0.138 | 0.790 | -0.307 | 0.234 | 1.607 |
|  | **Education level (Reference = Primary school)** |  |  | 0.554 |  |  |  |
|  | Junior high school vs Primary school | 0.146 | 0.147 | 0.320 | -0.142 | 0.433 | 2.266 |
|  | Senior high school or above vs Primary school | 0.058 | 0.157 | 0.714 | -0.251 | 0.366 | 2.788 |
|  | **Residence** |  |  |  |  |  |  |
|  | Urban vs Rural | 0.289 | 0.230 | 0.208 | -0.161 | 0.739 | 2.154 |
|  | **Living arrangement (Reference = With spouse)** |  |  | 0.026 |  |  |  |
|  | With children vs With spouse | -0.368 | 0.185 | 0.046 | -0.730 | -0.006 | 2.391 |
|  | Alone or with others vs With spouse | 0.014 | 0.277 | 0.961 | -0.528 | 0.556 | 2.453 |
| Environmental factors | Residential density | 0.057 | 0.052 | 0.273 | -0.045 | 0.159 | 1.730 |
| Behavioural cognitive factors | Lack of exercise equipment | 0.013 | 0.016 | 0.434 | -0.019 | 0.045 | 1.726 |
| **Model fit** | Robust Wald χ²=48.778, P<0.001 | | | | | | |

**Notes**. β coefficients are reported on the fractional-logit scale and should not be interpreted as odds ratios. HC3 robust standard errors and 95% confidence intervals are reported. For multicategory predictors, parent-row P values are from joint HC3 robust Wald tests, whereas category-row P values refer to individual contrasts with the reference category.

**Appendix 7.** Additional full-sample continuous screen-based sedentary proportion analysis (n=419)

| **Theoretical domain** | **Variable** | ***β coefficient*** | ***HC3 robust SE*** | ***P value*** | ***95% CI*** | | ***VIF*** |
| --- | --- | --- | --- | --- | --- | --- | --- |
| Personal factors | Age, years | -0.013 | 0.007 | 0.062 | -0.027 | 0.001 | 1.801 |
|  | BMI | -0.002 | 0.011 | 0.869 | -0.023 | 0.020 | 1.090 |
|  | 2hPG | 0.025 | 0.011 | 0.018 | 0.004 | 0.046 | 1.398 |
|  | HbA1c | -0.040 | 0.026 | 0.119 | -0.090 | 0.010 | 1.392 |
|  | Handgrip strength | -0.001 | 0.008 | 0.915 | -0.017 | 0.015 | 2.485 |
|  | **Five-times-sit-to-stand test (Reference = Higher performance)** |  |  | 0.683 |  |  |  |
|  | Lower performance vs Higher performance | 0.065 | 0.108 | 0.549 | -0.147 | 0.276 | 1.633 |
|  | Uncompleted vs Higher performance | 0.169 | 0.211 | 0.423 | -0.244 | 0.583 | 3.149 |
|  | **6-m gait speed (Reference = Higher performance)** |  |  | 0.014 |  |  |  |
|  | Lower performance vs Higher performance | -0.328 | 0.113 | 0.004 | -0.549 | -0.107 | 1.796 |
|  | Uncompleted vs Higher performance | -0.219 | 0.200 | 0.274 | -0.612 | 0.174 | 2.891 |
|  | **Subjective lower-limb weakness** |  |  |  |  |  |  |
|  | Yes vs No | 0.287 | 0.122 | 0.019 | 0.047 | 0.527 | 1.634 |
|  | **Gender** |  |  |  |  |  |  |
|  | Male vs Female | 0.020 | 0.112 | 0.861 | -0.200 | 0.239 | 1.752 |
|  | **Family history of diabetes** |  |  |  |  |  |  |
|  | Yes vs No | 0.223 | 0.086 | 0.010 | 0.053 | 0.392 | 1.050 |
|  | **Marital status** |  |  |  |  |  |  |
|  | Unmarried vs Married | 0.018 | 0.173 | 0.919 | -0.322 | 0.357 | 2.579 |
|  | **Monthly household income, CNY** |  |  |  |  |  |  |
|  | ≥2000 vs <2000 | 0.036 | 0.122 | 0.766 | -0.203 | 0.276 | 1.635 |
|  | **Education level (Reference = Primary school)** |  |  | 0.523 |  |  |  |
|  | Junior high school vs Primary school | 0.138 | 0.128 | 0.282 | -0.113 | 0.389 | 1.997 |
|  | Senior high school or above vs Primary school | 0.063 | 0.132 | 0.635 | -0.196 | 0.321 | 2.614 |
|  | **Residence** |  |  |  |  |  |  |
|  | Urban vs Rural | 0.321 | 0.176 | 0.069 | -0.025 | 0.667 | 2.442 |
|  | **Living arrangement (Reference = With spouse)** |  |  | 0.018 |  |  |  |
|  | With children vs With spouse | -0.298 | 0.138 | 0.031 | -0.569 | -0.027 | 1.901 |
|  | Alone or with others vs With spouse | 0.073 | 0.217 | 0.735 | -0.352 | 0.498 | 2.068 |
| Environmental factors | Residential density | 0.054 | 0.046 | 0.244 | -0.037 | 0.145 | 2.068 |
| Behavioural cognitive factors | Lack of exercise equipment | 0.020 | 0.014 | 0.132 | -0.006 | 0.047 | 1.496 |
| **Model fit** | Robust Wald χ²=91.907, P<0.001 | | | | | | |

**Notes.** β coefficients are reported on the fractional-logit scale and should not be interpreted as odds ratios. HC3 robust standard errors and 95% confidence intervals are reported. For multicategory predictors, parent-row P values are from joint HC3 robust Wald tests, whereas category-row P values refer to individual contrasts with the reference category.
